# From Hair to Colon: Hair Follicle-Derived MSCs Alleviate Pyroptosis in DSS-Induced Ulcerative Colitis by Releasing Exosomes in a Paracrine Manner

**DOI:** 10.1155/2022/9097530

**Published:** 2022-09-16

**Authors:** Yuan Chang, Yichi Zhang, Yanan Jiang, Lei Zhao, Chengqian Lv, Qianqian Huang, Jingming Guan, Shizhu Jin

**Affiliations:** ^1^Department of Gastroenterology and Hepatology, The Second Affiliated Hospital, Harbin Medical University, Harbin, Heilongjiang Province 150086, China; ^2^Department of Pharmacology (State-Province Key Laboratories of Biomedicine-Pharmaceutics of China, Key Laboratory of Cardiovascular Research, Ministry of Education), College of Pharmacy of Harbin Medical University, Harbin, Heilongjiang Province 150081, China

## Abstract

Ulcerative colitis (UC) has attracted intense attention due to its high recurrence rate and the difficulty of treatment. Pyroptosis has been suggested to be crucial in the development of UC. Although mesenchymal stem cells (MSCs) are broadly used for UC therapy, they have rarely been studied in the context of UC pyroptosis. Hair follicle-derived MSCs (HFMSCs) are especially understudied with regard to UC and pyroptosis. In this study, we aimed to discover the effects and potential mechanisms of HFMSCs in UC. We administered HFMSCs to dextran sulfate sodium- (DSS-) treated mice and found that the HFMSCs significantly inhibited pyroptosis to alleviate DSS-induced UC. A transwell system and GW4869, an exosome inhibitor, were used to prove the paracrine mechanism of HFMSCs. HFMSC supernatant reduced pyroptosis-related protein expression and promoted cell viability, but these effects were attenuated by GW4869, suggesting a role for HFMSC-released exosomes (Exos) in pyroptosis. Next, Exos were extracted and administered *in vitro* and *in vivo* to explore their roles in pyroptosis and UC. In addition, the biodistribution of Exos in mice was tracked using an imaging system and immunofluorescence. The results suggested that Exos not only improved DSS-induced pyroptosis and UC but also were internalized into the injured colon. Furthermore, the therapeutic efficacy of Exos was dose dependent. Among the Exo treatments, administration of 400 *μ*g of Exos per mouse twice a week exhibited the highest efficacy. The differentially expressed miRNAs (DEmiRNAs) between MSCs and MSC-released Exos suggested that Exos might inhibit pyroptosis through tumour necrosis factor-related apoptosis-inducing ligand (TRAIL) signalling and interferon- (IFN-) gamma pathways. Our study reveals that HFMSCs can alleviate pyroptosis in UC by releasing DEmiRNA-containing Exos in a paracrine manner. This finding may lead to new treatments for UC.

## 1. Introduction

Ulcerative colitis (UC), an immune-associated inflammatory disease, has attracted attention globally due to its increasing incidence and recurrence rates [1] and poses great threats to health care and the economy worldwide [2]. Investigating the pathogenesis of UC and identifying more effective treatments are popular research topics. Although the pathogenesis of UC remains underexplored, this condition is believed to be caused by an abnormal mucosal immune response and excessive inflammation in response to bacterial antigens [3]. The currently available medical treatments for UC, which include 5-aminosalicylic acid (5-ASA), hormones, immunosuppressants, and biological agents, have resulted in some improvements [4, 5]; however, due to their limited efficacy and complications, some patients still experience relapse and must consider surgical treatment [6]. Thus, more advanced treatments for UC are urgently needed.

Due to the anti-inflammatory and immunomodulatory abilities of mesenchymal stem cells (MSCs) [7, 8], transplantation of these cells has shown encouraging efficacy in UC [9–11]. Compared with MSCs from other sources, hair follicle-derived MSCs (HFMSCs) are more abundant, do not have age limitations, and are easier to obtain in a minimally invasive manner from patients [12]. HFMSCs also have much lower immunogenicity than other MSCs and do not have associated ethical issues [13]. In addition, a previous study has indicated that HFMSCs have greater proliferation ability than bone marrow MSCs [14]. Given these characteristics and the multidirectional differentiation potential of HFMSCs [13], these cells may have improved therapeutic prospects for UC.

MSCs have been demonstrated to exhibit homing, differentiation, and paracrine signalling abilities to exert anti-inflammatory and immunomodulatory effects [15]. However, recent studies have suggested that MSCs exert their effects through paracrine pathways rather than homing and differentiation pathways [15, 16]. MSC-released exosomes (Exos), which are particularly important paracrine components of MSCs, are significantly involved in intercellular signal communication [17]. Various proteins, mRNAs, miRNAs, and other molecules in MSC-released Exos are believed to influence the biological processes of target cells [18]. In particular, miRNAs in MSC-released Exos are strongly recommended for the treatment of UC [15, 19, 20]. Fully exploring the mechanisms of Exos may promote understanding of the functions of MSCs. Furthermore, given their substantial genetic material content, nonimmunogenicity, small size, and high transport efficiency [18], Exos may be promising treatment agents for UC.

Recent studies have shown that the nucleotide oligomerization domain- (NOD-) like receptor pyrin domain-containing protein 3 (NLRP3) inflammasome has vital impact on the immune and inflammatory responses of the intestinal mucosa in UC [21, 22]. The NLRP3 inflammasome is also the initial factor in pyroptosis that exacerbates UC [23]. Activated NLRP3 recruits the apoptosis-associated speck-like protein containing a caspase recruitment domain (ASC) protein, and the caspase-1 protein assembles into the inflammasome to cleave the caspase-1 protein and produce large amounts of interleukin-1*β* (IL-1*β*) and interleukin-18 (IL-18). Then, these proinflammatory cytokines are released through gasdermin D (GSDMD) to initiate pyroptosis in UC [24, 25]. A recent study has demonstrated that effectively suppressing NLRP3-induced pyroptosis can improve experimental colitis [26]. In addition, Cai et al. [27] reported that the NLRP3 inflammasome and pyroptosis pathway in UC can be blocked by MSC-derived Exos containing the miRNA 378a-5p. These findings indicate that NLRP3 and the pyroptosis pathway may be the targets of MSCs for UC treatment.

In this study, we applied HFMSCs in UC to evaluate the therapeutic effects and explore the potential mechanisms of HFMSCs. The results may lay a theoretical foundation for the application of HFMSCs in UC treatment.

## 2. Materials and Methods

### 2.1. Mice

C57BL/6J mice aged 6–8 weeks were obtained from the Animal Research Center of the Second Affiliated Hospital of Harbin Medical University. The animal experiments were carried out in strict accordance with the guidelines of the Ethics Committee of the Second Affiliated Hospital of Harbin Medical University (no. SYDW2019-252).

### 2.2. Isolation of HFMSCs

HFMSCs were isolated as previously described [28, 29]. The skin and hair follicles of healthy mice were collected for extraction of HFMSCs. After several rounds of disinfection, the tissues were cut into 3∗3 mm^2^ blocks and incubated with type I collagenase (0.1%, Sigma-Aldrich, USA) at 37°C for 1-2 h. Under a stereomicroscope, hair follicles were then extracted and placed into type I collagenase for 3-4 h and trypsin for 1 h. Foetal bovine serum (FBS, ScienCell, USA) was used for the neutralization of trypsin. HFMSCs were obtained from the neutralizing solution after being centrifuged at 1000 rpm for 10 min. The HFMSCs were plated in Dulbecco's modified Eagle's medium/F12 (DMEM/F12, Gibco, USA) containing 15% FBS and 1% penicillin–streptomycin (Gibco). After 10-14 days, the cells reached 70–80% confluence. The cells were then expanded to passages 2–5 for subsequent experiments.

### 2.3. Identification of HFMSCs

HFMSCs were characterized at passages 2–3. The positive MSC surface markers CD90 and CD29 and negative MSC surface markers CD31 and CD43 [29, 30] were detected by flow cytometry. The presence of an HFMSC-specific surface marker [31], cytokeratin 15 (CK15) (1 : 500, SC-47697, Santa Cruz), was verified by immunofluorescence. Alizarin red (Sigma-Aldrich) and Oil red O (Sigma-Aldrich) were used to identify the effects of HFMSCs on osteogenic and adipogenic differentiation. All procedures were conducted as previously described [32].

### 2.4. Establishment and Evaluation of Dextran Sulfate Sodium- (DSS-) Induced UC

To establish a UC animal model as previously described [33], mice were given 2.5% DSS (MW = 36, 000 − 50,000 Da; MP Biomedicals, Canada) dissolved in drinking water and allowed to drink freely for 7 days and then to recover for 3 days. All mice were randomly divided into 4 groups: the control group (*n* = 12), the DSS+PBS group (*n* = 12), the DSS+HFMSC group (*n* = 6), and the DSS+Exo group (*n* = 18). Each mouse in the HFMSC treatment group was treated with 3 × 10^6^ HFMSCs via the tail vein on the 3^rd^ day. The mice in the DSS+Exo group were administered Exos via the tail vein on the 3^rd^ and 5^th^ days. The DSS+Exo group was divided into three subgroups for administration of different doses of Exos; 100 *μ*g, 200 *μ*g, and 400 *μ*g of Exos per mouse were injected twice a week into the mice in the DSS+Exo^1^, DSS+Exo^2^, and DSS+Exo^3^ groups, respectively.

To assess the severity of UC [34], body weight loss, diarrhoea, and bloody stool were recorded daily. The disease activity index (DAI) was evaluated according to previously described methods [34]. On the 11^th^ day, the mice were sacrificed. The colon length of each mouse (from the rectum to the caecum) was measured and analysed. Histological assessment of colitis caused by epithelial injury and inflammatory infiltration was performed via haematoxylin and eosin (HE) staining [35].

### 2.5. Coculture of HFMSCs or Exos with MODE-K Cells

MODE-K, a mouse intestinal epithelial cell line obtained from Shenzhen Huatuo Biological Engineering (Shenzhen, China), was used in this study. For detection of uptake by MODE-K cells, HFMSCs or Exos labelled with PKH67 using a fluorescence labelling kit (Sigma-Aldrich, USA) were cocultured with MODE-K cells in a transwell system for 24 h. All fluorescence images were acquired by fluorescence microscopy. MODE-K cells were stimulated to induce NLRP3 inflammasome activity with 200 ng/ml lipopolysaccharide (LPS) (Sigma-Aldrich, USA) for 4 h and then 5 mM adenosine 5-triphosphate (ATP) (SunShine Biotechnology, China) for 30 min. GW4869 (10 *μ*M, Selleck, USA) solubilized in 0.1% DMSO was diluted in culture medium and then applied to verify the paracrine effects of HFMSCs. Exos (100 *μ*g/ml) were incubated with LPS+ATP for treatment of MODE-K cells. The cells were subjected to different stimuli and divided into the following groups: the control group, the LPS+ATP group, the LPS+ATP+DMSO group, the LPS+ATP+GW4869 group, the LPS+ATP+HFMSC supernatant (LPS+ATP+HFMSC) group, the LPS+ATP+HFMSC supernatant+GW4869 (LPS+ATP+HFMSC+GW4869) group, and the LPS+ATP+Exo group. After incubation for 4.5 h, the MODE-K cells and the cell supernatant were collected for subsequent experiments.

### 2.6. HE Staining

Mouse colons were fixed with 4% paraformaldehyde, dehydrated with alcohol, cleared with xylene, embedded in paraffin, and cut into 5 *μ*m sections. HE staining was carried out as described previously [15].

### 2.7. Immunohistochemistry and Immunofluorescence Staining

Colon tissues were sectioned into 4 *μ*m sections. Immunohistochemistry was performed, and the results were analysed as described above [36]. The immunohistochemistry images were obtained with an Olympus (BX41) microscope and semiquantitatively analysed with Fiji software.

Exos labelled with PKH67 were administered to the mice in the DSS+Exo^3^ group, and frozen colon sections from the DSS+Exo^3^ group were subjected to immunofluorescence staining. Immunofluorescence staining was carried out as described previously [36]. Immunofluorescence images were then collected with a fluorescence microscope (Zeiss-DMI8).

All primary antibodies are presented in Table S1.

### 2.8. Western Blotting

Colons and cell samples were lysed for extraction of proteins, and the BCA method was applied to measure the protein concentration. Western blotting was implemented as previously described [15]. Images of the protein bands were then collected using an ImageQuant LAS 334 4000 mini machine (GE). The primary antibodies are shown in Table S1.

### 2.9. Enzyme-Linked Immunosorbent Assay (ELISA)

Mouse venous blood and the supernatant of MODE-K cells were collected and centrifuged. ELISA was used to examine the levels of IL-1*β* and IL-18 in the samples. All experimental procedures were performed according to the protocols provided with the ELISA kits (Boster, China).

### 2.10. Cell Viability Analysis

Cell viability was assessed with a Cell Counting Kit-8 (CCK-8) (APExBIO-K1018) and 5-ethynyl-2′-deoxyuridine (EdU) imaging kits (UE, China). A total of 2 × 10^3^ MODE-K cells per well were cultured in 96-well plates and treated as described above. The CCK-8 and EdU assays were performed according to the manufacturer's recommended procedures.

### 2.11. Tracking of Labelled Exos *In Vivo*

Exos were labelled with 1,1′-dioctadecyl-3,3,3′,3′-tetramethylindotricarbocyanine iodide (DiR; Thermo Fisher Scientific/Invitrogen, USA), and DiR-labelled Exos (Exos^DiR^, 400 *μ*g per mouse) were administered to healthy mice and DSS-treated mice. Images of the mice and tissues were obtained 24 h after the administration of Exo^DiR^ using an X spectral imaging instrument and *in vivo* imaging software (NightOWL II LB983).

### 2.12. Differentially Expressed miRNA (DEmiRNA) Analysis and Functional Enrichment Analysis

From the Gene Expression Omnibus (GEO) database, we obtained the dataset GSE71241 (https://www.ncbi.nlm.nih.gov/geo/query/acc.cgi?acc=GSE71241) of miRNAs in human MSC-released Exos. Exos derived from MSCs were used for the experimental group (EXO, *n* = 9). MSC samples were used for the control group (Control, *n* = 9). DEmiRNAs were identified from the EXO and Control groups using the limma package with the criteria of an adjusted *P* value < 0.05 and a |log2FC| value > 1. Functional enrichment of the DEmiRNAs was performed via Kyoto Encyclopedia of Genes and Genomes (KEGG) and Gene Ontology (GO) analyses using FUNRICH software to recognize the cellular components (CCs), molecular functions (MFs), biological processes (BPs), and related biological pathways involved. The upregulated DEmiRNAs were sorted by their |log2FC| values, and the top 5 miRNAs were used to predict the target genes using the miRTarBase database.

### 2.13. Statistical Analysis

All experiments were carried out three times. The data are shown as the means ± SDs and were analysed by one-way analysis of variance (ANOVA) with GraphPad Prism 8.0. Values of *P* < 0.05 were considered to indicate significance.

## 3. Results

### 3.1. HFMSCs Attenuated DSS-Induced UC

**Figure 1:**
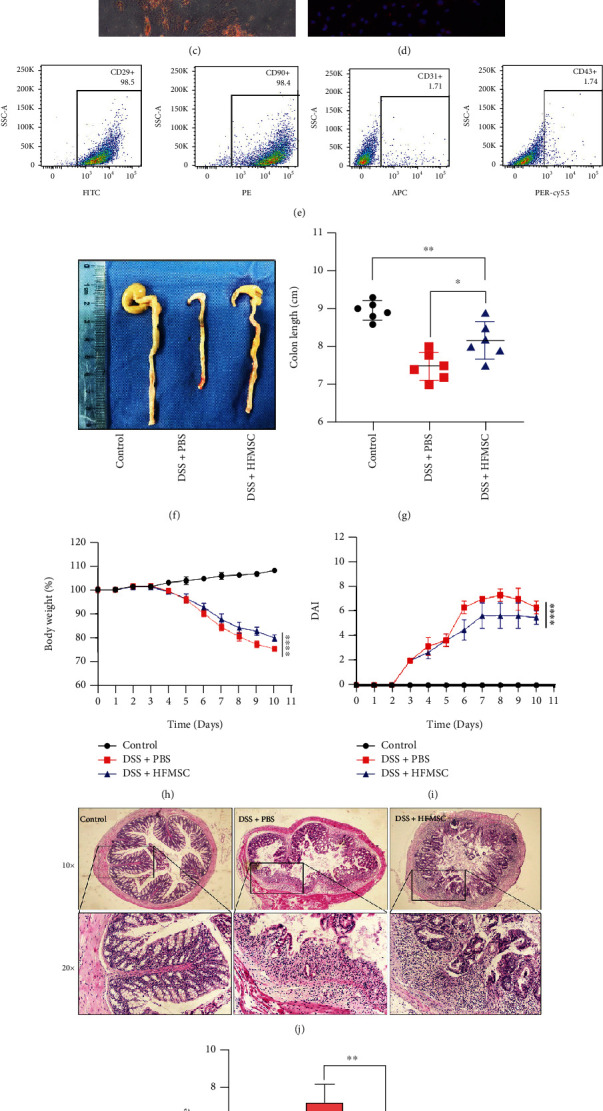
Investigation of the effects of HFMSCs on DSS-induced UC. (a) The morphology of HFMSCs was observed under a white-light microscope. (b, c) HFMSCs stained with Alizarin red and Oil red O differentiated into osteoblasts and adipocytes. (d) CK15 was observed by immunofluorescence. (e) Surface markers of HFMSCs, including positive markers (CD90 and CD29) and negative markers (CD31 and CD43), were detected by flow cytometry. HFMSCs were administered to DSS-treated mice. (f, g) The colon lengths of the mice in all the groups were measured and analysed. (h, i) The body weight loss and DAI values were compared among the three groups. (j) The colon histology of the mice in the three groups was detected by HE staining. (k) Mucosal damage and inflammatory infiltration in the colons were determined. All the data are displayed as the means ± SDs. ^∗^*P* < 0.05, ^∗∗^*P* < 0.01, and ^∗∗∗^*P* < 0.001. ns: not significant. The images were obtained with 10x and 20x Olympus dotSlide objectives.

HFMSCs were isolated from mouse hair follicles as described above [28, 29]. Under a white light microscope, HFMSCs were observed as adherent cells exhibiting spindle-like shapes (Figure 1(a)). As shown in Figures 1(b) and 1(c), HFMSCs stained by Alizarin red and Oil red O differentiated into osteoblasts and adipocytes. Immunofluorescence revealed that the HFMSC-specific protein CK15 was concentrated on the cell membrane (Figure 1(d)). Flow cytometry showed high expression of positive surface markers of MSCs (CD29: 98.5%, CD90: 98.4%) and low expression of negative surface markers of MSCs (CD31: 1.71%, CD43: 1.74%) (Figure 1(e)). Thus, HFMSCs were confirmed to be MSCs derived from hair follicles. On the 3^rd^ day, the mice began to develop diarrhoea, weight loss, and bloody stool, and these symptoms persisted until the end of the DSS intervention. The morphology and length of the mouse colons in the three groups were assessed and compared on the 11^th^ day (Figure 1(f)). The colon length in the HFMSC treatment group was greater than that in the model group but less than that in the control group (Figure 1(g)). Based on the symptoms recorded daily for 10 days, the model group treated with DSS+PBS presented a significantly decreased body weight and elevated DAI values. However, these changes were markedly improved in the HFMSC treatment group (Figures 1(h) and 1(i)). Furthermore, as demonstrated by the histological analysis of the colon, the DSS+HFMSC group showed obviously decreased mucosal damage and clearly lower inflammatory infiltration than the DSS+PBS group (Figures 1(j) and 1(k)). Together, these results confirmed that HFMSCs substantially alleviated DSS-induced UC.

### 3.2. HFMSCs Reduced Pyroptosis to Relieve DSS-Induced UC

**Figure 2:**
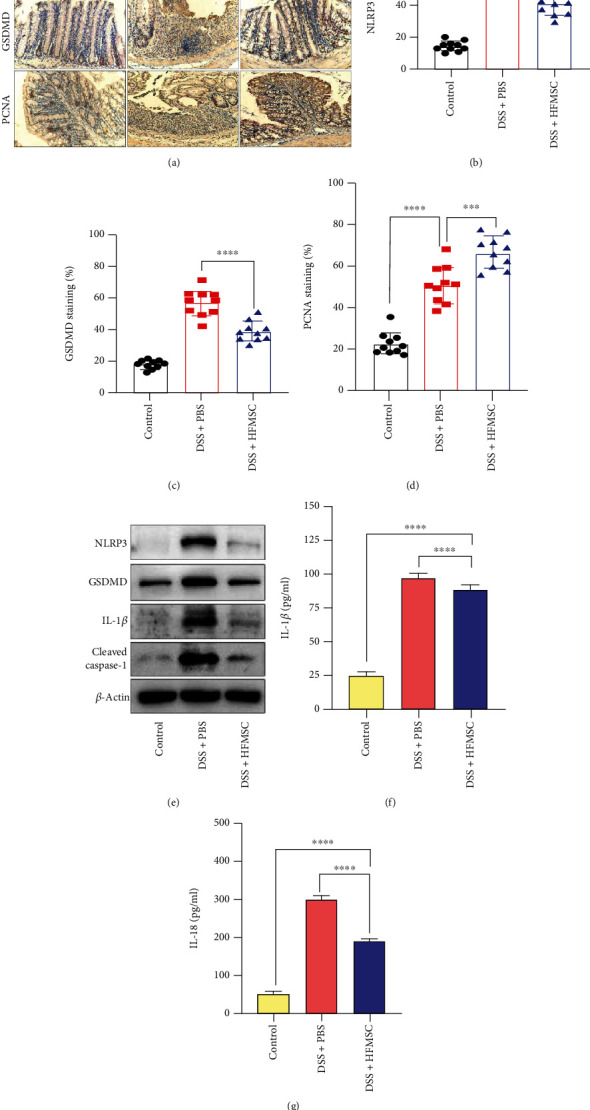
HFMSCs reduced pyroptosis *in vivo*. To detect the impact of HFMSCs on pyroptosis in DSS-treated mice, (a) the expression of the NLRP3, GSDMD, and PCNA proteins was detected by immunohistochemistry. (b–d) The efficacy of HFMSCs in the model mice was assessed semiquantitatively. (e) Western blotting was carried out to examine the protein expression of NLRP3, GSDMD, cleaved caspase-1, and IL-1*β*. In addition, (f, g) ELISA was applied to test the protein levels of IL-1*β* and IL-18 in the serum. All the data are displayed as the means ± SDs. ^∗^*P* < 0.05, ^∗∗^*P* < 0.01, and ^∗∗∗^*P* < 0.001. ns: not significant. The images were obtained with an Olympus dotSlide objective. Scale bar: 100 *μ*m.

NLRP3 inflammasome-induced pyroptosis has been proven to play a key role in UC [21, 22]. To identify the effect of HFMSCs on pyroptosis, the protein expression of NLRP3, GSDMD, and proliferating cell nuclear antigen (PCNA) was detected by immunohistochemistry (Figure 2(a)). According to the semiquantitative analysis, the DSS+PBS group showed the highest number of positively stained cells with the NLRP3 and GSDMD proteins, while the staining for these proteins was distinctly reduced in the HFMSC treatment group (Figures 2(b) and 2(c)). In addition, the DSS+HFMSC group presented the highest numbers of PCNA-stained cells among the three groups (Figure 2(d)). To further prove the ability of HFMSCs to inhibit pyroptosis, western blotting and ELISA were used to detect the levels of pyroptosis-related proteins. Lower protein levels of NLRP3, GSDMD, cleaved caspase-1, and IL-1*β* were observed in the HFMSC treatment group than in the model group, as shown in Figure 2(e). Statistical analysis of these protein levels is exhibited in Figure S1. ELISA revealed that HFMSCs markedly reduced the protein levels of IL-1*β* and IL-18 (Figures 2(f) and 2(g)). Thus, we concluded that HFMSCs could distinctly suppress pyroptosis in UC.

### 3.3. HFMSCs Inhibited Pyroptosis *In Vitro* in a Paracrine Manner

**Figure 3:**
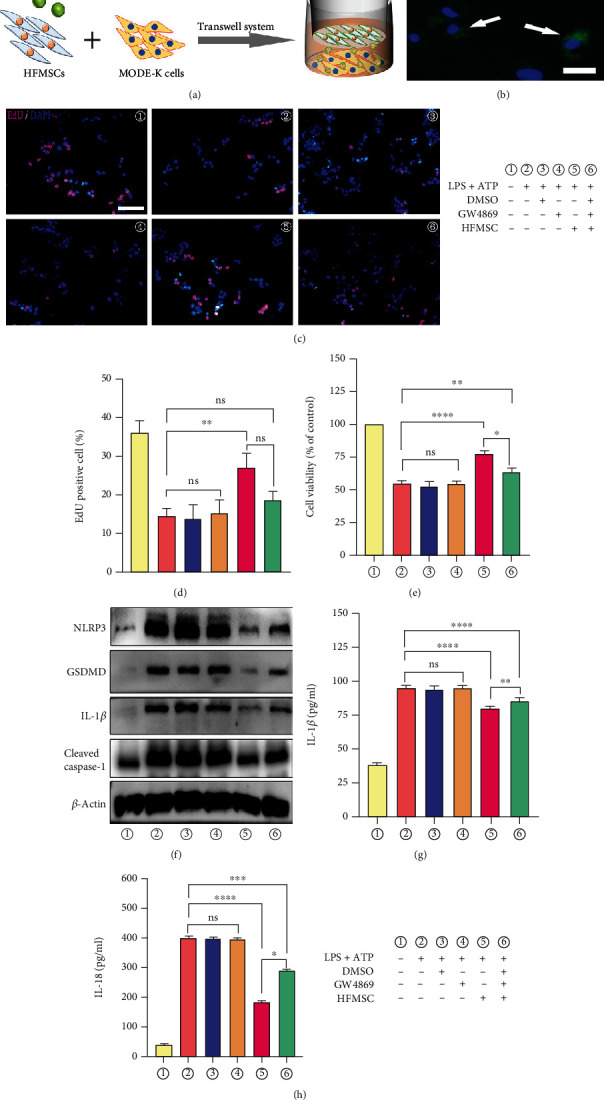
HFMSCs exerted a protective effect against pyroptosis in a paracrine manner. (a) After PKH67 staining, HFMSCs were cocultured with MODE-K cells in a transwell system. (b) The fluorescence of PKH67 was then observed by fluorescence microscopy to detect the uptake of HFMSCs by MODE-K cells in a paracrine manner. Scale bar, 50 *μ*m. MODE-K cells were divided into six groups for different treatments. (c–e) The cell viability in each group was determined through EdU and CCK-8 assays. Scale bar, 200 *μ*m. (f) The results of western blotting revealed the levels of pyroptosis-related proteins in all the groups. (g, h) ELISA was used to analyse the IL-1*β* and IL-18 protein levels in the supernatant of each group. All the data are displayed as the means ± SDs. ^∗^*P* < 0.05, ^∗∗^*P* < 0.01, and ^∗∗∗^*P* < 0.001. ns: not significant.

Recent studies have confirmed that the paracrine mechanism of MSCs is crucial and effective in many diseases [15, 37, 38]. To discover the role of the paracrine pathway in the effects of HFMSCs, a transwell system was implemented. In this system, MODE-K cells were plated in the lower chamber and cocultured with PKH67-labelled HFMSCs in the upper chamber (Figure 3(a)). After 24 h, marked green fluorescence in MODE-K cells was detected using fluorescence microscopy, which indicated the paracrine uptake of HFMSC components by MODE-K cells (Figure 3(b)). It is well known that Exos are a main component of the paracrine pathway of MSCs. To further verify the paracrine mechanism of HFMSCs through Exo release, we cocultured HFMSCs with GW4869, which can inhibit Exo generation and release. The supernatant of HFMSCs cultured with or without GW4869 was incubated with MODE-K cells stimulated with LPS+ATP for 4.5 h. As shown in Figures 3(c)–3(e), the supernatant of HFMSCs markedly increased the viability of MODE-K cells; the next-greatest viability was observed in the LPS+ATP+HFMSC+GW4869 group. In addition, no significant differences were found among the LPS+ATP group, the DMSO treatment group, and the GW4869 treatment group. Although the supernatant of HFMSCs effectively protected cells from pyroptosis, this protection was attenuated by GW4869, which implies that Exos are crucial for the paracrine pathway of HFMSCs. Furthermore, western blotting and ELISA were used to test the levels of pyroptosis-related proteins in all the groups. Compared with those in the model group, the levels of the proteins NLRP3, GSDMD, cleaved caspase-1, IL-1*β*, and IL-18 were prominently reduced in the LPS+ATP+HFMSC group and decreased to a lesser extent in the LPS+ATP+HFMSC+GW4869 group (Figures 3(f)–3(h) and Figure S2). There were still no significant differences among the LPS+ATP group, the DMSO treatment group, and the GW4869 treatment group. The abovementioned findings demonstrated that HFMSCs attenuated pyroptosis in a paracrine manner by releasing Exos.

### 3.4. HFMSC-Released Exos Attenuated Pyroptosis *In Vitro*

We extracted Exos by differential centrifugation. The typical cup- and sphere-shaped morphologies were observed by TEM (Figure S3A). NTA results demonstrated that the Exo particle size was approximately 90 nm (Figure S3B). The surface proteins CD9 and TSG101 were enriched in Exos compared with HFMSCs, whereas the protein calnexin was rarely expressed in Exos, as indicated by western blotting (Figure S3C). Considering the uptake of HFMSCs by MODE-K cells in the transwell system, we detected the internalization of PKH67-labelled Exos by MODE-K cells using immunofluorescence. Green fluorescence appeared in MODE-K cells after incubation with Exos (Figure S3D), which implied that the HFMSC components were internalized by MODE-K cells via released Exos. To further verify the effect of the supernatant of HFMSCs on pyroptosis through Exos, Exos were applied to inhibit pyroptosis *in vitro*. As evidenced by EdU and CCK-8 assays, Exos markedly promoted the viability of MODE-K cells compared with that in the LPS+ATP group (Figures S3E–S3G). Western blotting demonstrated that Exos significantly inhibited pyroptosis by decreasing the protein levels of NLRP3, GSDMD, cleaved caspase-1, and IL-1*β* (Figure S3H). Moreover, the expression of the proteins IL-1*β* and IL-18 in the supernatant of MODE-K cells treated with Exos was lower than that in the LPS+ATP group, as shown by ELISA (Figures S3I and S3J). In general, these results confirmed that Exos could effectively limit pyroptosis *in vitro* and were key components of HFMSCs.

### 3.5. Exos Exerted a Therapeutic Effect *In Vivo*

**Figure 4:**
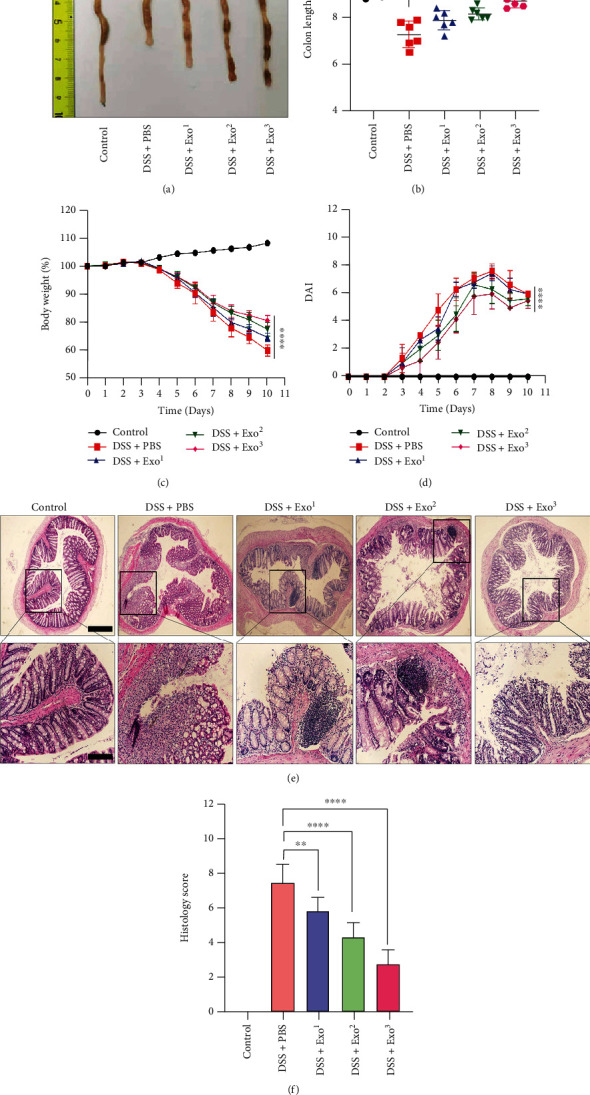
Exos exerted a therapeutic effect *in vivo*. Because Exos inhibited pyroptosis *in vitro*, we hypothesized that Exos could also be effective *in vivo*. C57BL/6J mice administered with DSS were treated with different doses of Exos (Exo^1^: 100 *μ*g, Exo^2^: 200 *μ*g, and Exo^3^: 400 *μ*g; twice a week for each mouse). (a) The colons of the mice were extracted, and (b) the colon lengths were compared. (c, d) The body weights and DAI values of the mice in each group were recorded and compared. (e) HE staining was performed to assess colon histology in each group. (f) After comparing the images of HE staining among the five groups, the histology scores with intestinal mucosa ulceration and inflammation were analysed. All the data are displayed as the means ± SDs. ^∗^*P* < 0.05, ^∗∗^*P* < 0.01, and ^∗∗∗^*P* < 0.001. ns: not significant. All images were obtained with an Olympus dotSlide objective. Scale bars: 200 *μ*m, 100 *μ*m.

Based on the therapeutic effect of Exos *in vitro*, we hypothesized that Exos could also be effective *in vivo*. C57BL/6J mice treated with DSS for 7 days were administered different doses of Exos (Exo^1^: 100 *μ*g, Exo^2^: 200 *μ*g, and Exo^3^: 400 *μ*g for each mouse) on the 3^rd^ and 5^th^ days. The colons of the mice in the five groups were measured and analysed on the 11^th^ day. In Figures 4(a) and 4(b), the colon lengths in the Exo^2^ and Exo^3^ treatment groups were clearly greater than those in the model group and the DSS+Exo^1^ group, whereas there was no significant difference in colon length between the Exo^2^ and Exo^3^ treatment groups. A significant difference was also not observed between the model group and the DSS+Exo^1^ group. After DSS administration for 3 days, the body weight of the mice, particularly in the model group, distinctly decreased over time. However, as shown in Figure 4(c), the Exo treatment groups exhibited less weight loss with increasing doses of Exos, although the data for the Exo^3^ and Exo^2^ treatment groups did not differ. As shown in Figure 4(d), the DAI scores in the Exo^3^ treatment group were significantly lower than those in the model group, followed by those in the DSS+Exo^2^ group. However, the DAI scores in the Exo^1^ treatment group did not significantly differ from those in the model group. Intestinal mucosal ulceration and inflammation gradually improved with increasing doses of Exos (Figures 4(e) and 4(f)). Based on the abovementioned results, we concluded that Exos relieved DSS-induced UC in a dose-dependent manner. Furthermore, we found that twice-weekly injection of 400 *μ*g of Exos was the most appropriate dose for alleviation of DSS-induced UC.

### 3.6. Exos Protected DSS-Treated Mice from Pyroptosis

**Figure 5:**
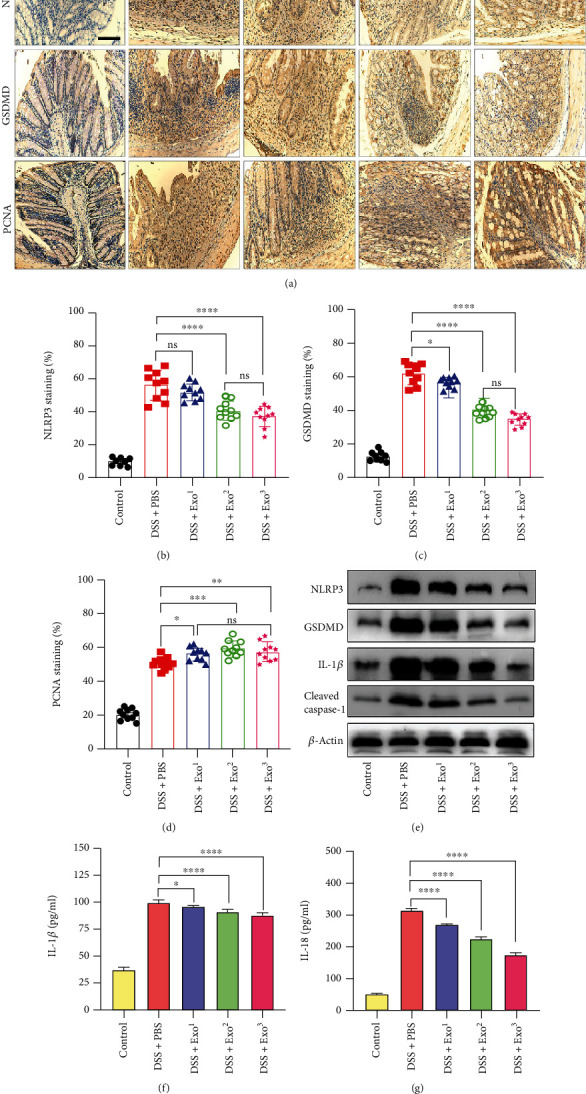
Exos relieved pyroptosis *in vivo*. (a–d) Immunohistochemistry and semiquantitative analysis revealed the protein levels of NLRP3, GSDMD, and PCNA in each group. (e–g) Western blotting and ELISA were applied to test the levels of pyroptosis-related proteins in the five groups. All the data are presented as the means ± SDs. ^∗^*P* < 0.05, ^∗∗^*P* < 0.01, and ^∗∗∗^*P* < 0.001. ns: not significant. The images were obtained with an Olympus dotSlide objective. Scale bar: 100 *μ*m.

We performed immunohistochemical staining of the colons from each group. As shown in Figures 5(a)–5(c), decreasing numbers of NLRP3- and GSDMD-positive cells were detected with increasing doses of Exo, and the lowest numbers were found in the DSS+Exo^2^ group and the DSS+Exo^3^ group. Additionally, the analysis of NLRP3-positive cells showed no clear distinction between the model group and the Exo^1^ treatment group. In contrast, increased numbers of PCNA-positive cells were detected in the Exo treatment groups (Figure 5(d)). Interestingly, the numbers of PCNA-positive cells did not significantly differ among the Exo treatment groups. Western blotting revealed that the NLRP3, GSDMD, cleaved caspase-1, and IL-1*β* protein levels decreased along with increasing Exo doses (Figure 5(e)). Statistical analysis of these protein levels was shown in Figure S4. The ELISA also showed that the protein expression of IL-1*β* and IL-18 was negatively correlated with the dose of Exos (Figures 5(f) and 5(g)). Accordingly, the above results indicated that Exos prominently blocked pyroptosis in a dose-dependent manner and promoted regeneration to some extent.

### 3.7. Exos Entered the Damaged Colon and Influenced Pyroptosis

**Figure 6:**
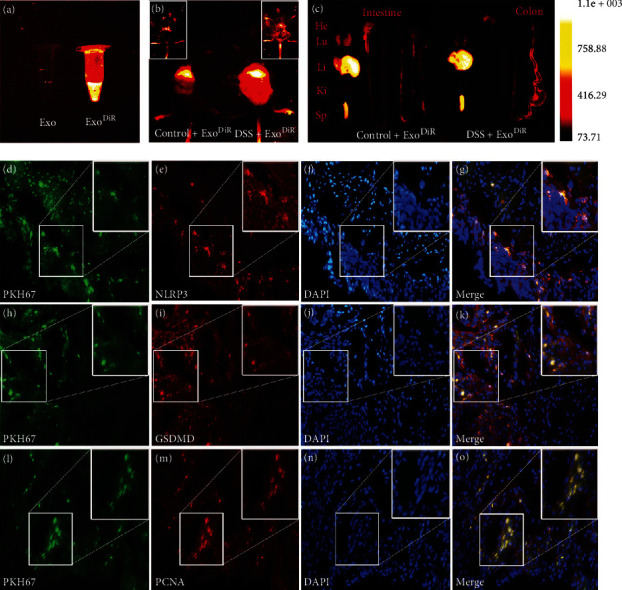
Distribution of Exos *in vivo*. To ensure the internalization of Exos *in vivo*, (a) DiR-labelled Exos were administered to healthy mice and DSS-treated mice. (b) Twenty-four hours after DSS administration, the fluorescence intensity was compared between the two groups. (c) The fluorescence accumulation in the heart, lungs, liver, kidneys, spleen, and intestine was detected. Moreover, (d–o) immunofluorescence revealed colocalization of Exos and the NLRP3, GSDMD, and PCNA proteins. The fluorescence images were obtained with an X spectral imaging instrument and *in vivo* imaging software (NightOWL II LB983). Immunofluorescence images were captured by 40x fluorescence microscopy (Zeiss-DMI8). Scale bar: 200 *μ*m.

Based on the effects of Exos in reducing pyroptosis, we attempted to determine the biological distribution of Exos. Previous studies have demonstrated that Exos infused into mice with colitis can reach the damaged colon and improve colitis [39, 40]. Accordingly, considering the uptake of Exos *in vitro*, DiR-labelled Exos were injected into healthy mice and DSS-treated mice, and the internalization of the Exos was examined *in vivo* (Figure 6(a)). After 24 h, the DiR dye was highly concentrated in the livers and spleens of mice in both groups. In addition, the colons of the mice in the DSS+Exo^DiR^ group presented clear DiR fluorescence (Figures 6(b) and 6(c)), which suggested that the Exos were internalized into the damaged colon, in line with the findings of previous studies [39, 40]. Moreover, considering the ability of Exos to reduce pyroptosis, we tracked the distributions of PKH67-labelled Exos in the DSS+Exo^3^ group by immunofluorescence. As shown in Figures 6(d)–6(o), Exos appeared at the same site as the NLRP3, GSDMD, and PCNA proteins, which further implied that Exos might target the injured colon to improve pyroptosis and promote regeneration *in vivo*.

### 3.8. Bioinformatics Analysis of miRNAs in MSC-Released Exos

**Figure 7:**
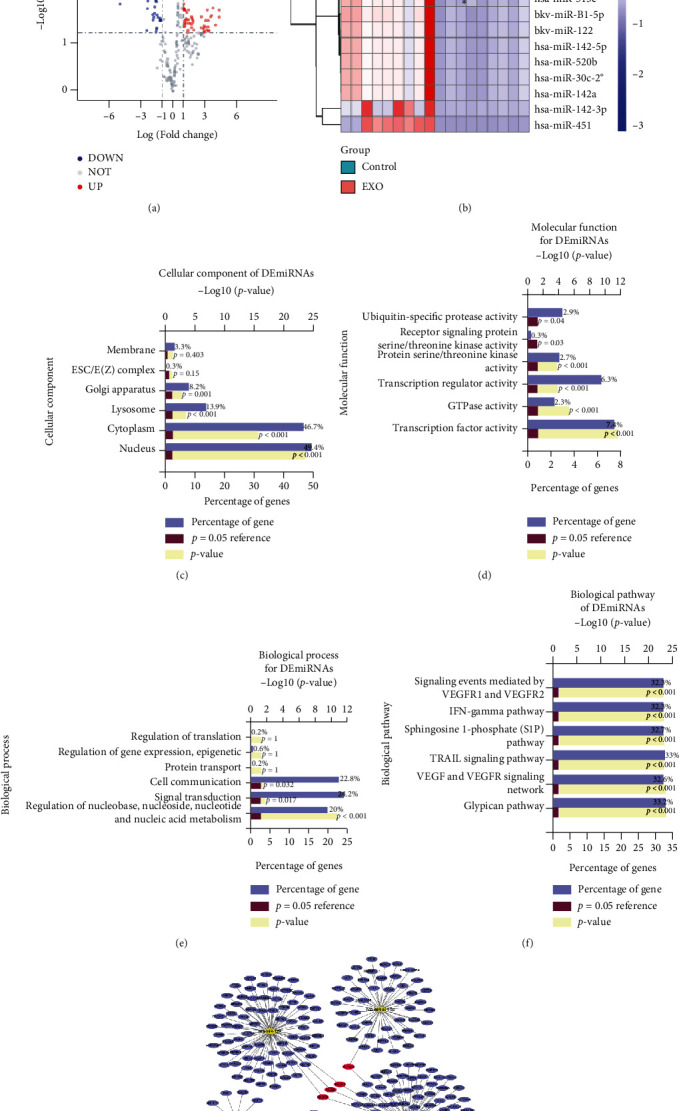
Differential expression and bioinformatics analysis of miRNAs in Exos derived from MSCs. (a) The DEmiRNAs between the EXO group and the control group are shown in a volcano plot, and (b) the differential expression of the top 10 upregulated DEmiRNAs and the top 10 downregulated DEmiRNAs is shown in a heat map. Functional enrichment analysis was performed to examine the (c) CC terms, (d) MF terms, (e) BP terms, and (f) biological pathways for the DEmiRNAs. (g) DEmiRNA-mRNA regulatory network with the top 5 upregulated DEmiRNAs.

To further explore the potential mechanism of MSC-released Exos, we selected the GSE71241 dataset from the GEO database and analysed the DEmiRNAs and their functional enrichment in MSC-released Exos. A total of 177 DEmiRNAs were identified in the EXO and Control groups according to the criteria of an adjusted *P* value < 0.05 and a |log2FC| value > 1, including 55 upregulated miRNAs and 122 downregulated miRNAs (Table S2). The DEmiRNAs are shown in the volcano plot (Figure 7(a)), and the heat map shows the top 10 upregulated DEmiRNAs (hsa-miR-520e, hsa-miR-513c, bkv-miR-B1-5p, hsa-miR-122, hsa-miR-142-5p, hsa-miR-520b, hsa-miR-30c-2∗, hsa-miR-422a, hsa-miR-142-3p, and hsa-miR-451) and top 10 downregulated DEmiRNAs (hsa-miR-196a, hsa-miR-193a-5p, hsa-miR-31∗, hsa-miR-574-3p, hsa-miR-654-3p, hsa-miR-210, hsa-miR-10a, hsa-miR-377, hsa-miR-125a-5p, and hsa-miR-143) in MSC-released Exos (Figure 7(b)). Functional enrichment analysis of the DEmiRNAs was further performed with the GO and KEGG databases. As shown in Figure 7(c), most DEmiRNAs were derived from the nucleus and cytoplasm. The MFs of the DEmiRNAs focused on transcription factor activity and transcription regulatory activity (Figure 7(d)). The results of BP enrichment indicated that the DEmiRNAs were involved in cell communication, signal transduction, and regulation of nucleobase, nucleoside, nucleotide, and nucleic acid metabolism (Figure 7(e)). KEGG biological pathway analysis demonstrated that tumour necrosis factor-related apoptosis-inducing ligand (TRAIL) signalling, interferon- (IFN-) gamma pathways, etc. were associated with the DEmiRNAs (Figure 7(f)). Notably, the TRAIL signalling and IFN-gamma pathways have been proven to limit receptor-interacting protein kinase 1 (RIPK1), mechanistic target of rapamycin (mTOR), and mixed-lineage kinase domain-like (MLKL) proteins to inhibit pyroptosis [41–43]. Furthermore, the miRTarBase database was used to predict the target genes of the top 5 upregulated DEmiRNAs (Figure 7(g)). According to the results of bioinformatics analysis, we hypothesize that Exos containing DEmiRNAs may inhibit pyroptosis through the TRAIL signalling and IFN-gamma pathways.

## 4. Discussion

In this study, we proved that HFMSCs could effectively relieve DSS-induced UC and pyroptosis by releasing Exos. Furthermore, our bioinformatics results indicated that the TRAIL and IFN-gamma signalling pathways may be involved in the effects of Exo treatment on pyroptosis. Our findings reveal the efficacy and mechanism of HFMSCs against UC, potentially providing a promising treatment for UC.

Hair follicles, as natural reservoirs of MSCs, are recognized as having advantages over other sources of MSCs [13]. In previous studies, HFMSCs have been suggested to be effective in the treatment of hair loss [30], pancreatitis [44], and liver cirrhosis [45] due to their anti-inflammatory properties and their ability to differentiate into parenchymal cells. However, there have been few studies on HFMSCs in the context of UC. In this study, HFMSCs were revealed to diminish colon shortening, body weight loss, bleeding, and colon injury, in line with the effects of MSCs derived from other sources on UC [9–11]. It has been shown that dental pulp MSCs transfected with hepatocyte growth factor can transdifferentiate into intestinal stem cells to reduce inflammation and restore mucosal integrity in UC [9]. However, Sala et al. [10] reported that the anti-inflammatory ability of MSCs in UC is more dependent on paracrine release of tumour necrosis factor-induced protein 6 than on homing to the damaged colon. Recently, the paracrine function of MSCs was proven to be closely related to the release of Exos [46, 47]. After GW4869 treatment, the effects of MSCs have been found to prominently decrease, consistent with our findings [47]. Studies on the application of Exos have further proven the key role of Exos in MSCs. Exos derived from human umbilical cord MSCs are believed to release miRNA 378a-5p to macrophages, potentially targeting NLRP3 to attenuate colitis [27]. Adipose MSC-released Exos have been suggested to regulate Foxp3+ Treg cells in the spleen and lymph nodes to reduce the inflammatory cytokine release induced by DSS [48]. As shown in our findings, NLRP3-induced pyroptosis was significantly inhibited by Exos, improving DSS-induced UC. Collectively, the evidence suggests that MSC-released Exos can modulate immune responses and control inflammatory responses to attenuate UC [49].

NLRP3 has been suggested to be the key protein leading to severe inflammation and the pyroptosis pathway in UC [21, 22]. A previous study has demonstrated that NLRP3^−/−^mice treated with DSS are not susceptible to UC and have lower levels of IL1*β* and IL18 than wild-type mice [50]. Pterostilbene derivatives have been revealed to improve experimental colitis by suppressing NLRP3-induced pyroptosis [26]. Another study has suggested that MSC-derived exosomal miR-378a-5p can target the mRNA of NLRP3 to inhibit pyroptosis and attenuate UC [27]. Gu et al. [51] reported that MSC-derived exosomal miR-181a limits the expression of proinflammatory factors (TNF-*α*, IL-6, IL-1*β*, IL-17, and IL-18) and improves epithelial integrity in UC. NLRP3 and NLRP3-induced pyroptosis may become therapeutic targets for UC. In addition, PCNA was found to be highly expressed in the HFMSC and Exo treatment groups, which implied that some potential functions of HFMSCs and Exos need to be explored in the future.

Emerging studies have demonstrated that miRNAs are highly enriched in Exos and perform multiple biological functions [20, 38]. We did not detect the levels of miRNAs in Exos but used bioinformatics tools to analyse the DEmiRNAs and their enriched functions. The TRAIL signalling pathway and IFN-gamma pathway were revealed to be enriched for the DEmiRNAs. Interestingly, these pathways have been demonstrated to positively regulate the NLRP3 inflammasome through the protein MLKL [42]. The protein TRAIL has also been revealed to interact with DR5 and NF*κ*B to stimulate the NLRP3 pathway [52, 53]. Another study has proven that IFN-gamma can inhibit mitochondrial integrity in Paneth cells by inducing mTORC1-dependent pyroptosis and stimulating intestinal inflammation [41]. Together, the evidence suggests that DEmiRNAs in MSC-released Exos may reduce pyroptosis by targeting the TRAIL signalling and IFN-gamma pathways, which should be further verified in our future research.

There is no clear standard for the dosage of MSC-released Exos for UC treatment. It has been reported that 200 *μ*g of bone marrow MSC-released Exos can protect mice that drink 5% DSS for 7 days from developing severe colitis [54]. Another study has demonstrated that three injections of 400 *μ*g/mouse human umbilical cord MSC-released Exos can downregulate IL-1*β* protein levels in mice with UC induced by treatment with 3% DSS for 11 days [39]. Treatment of mice with two injections of 60 *μ*g of olfactory/ecto-MSC-released Exos can alleviate 2.5% DSS-induced acute UC [19]. We hypothesize that the different effective frequencies and doses of MSC-released Exos may be attributable to the different sources of MSCs and the different DSS interventions. In our study, three Exo treatment groups (Exo^1^: 100 *μ*g, Exo^2^: 200 *μ*g, and Exo^3^: 400 *μ*g; twice a week in each mouse) were used to determine the appropriate dose of Exos. Based on the excellent amelioration of UC and pyroptosis, twice-weekly injection of 400 *μ*g of Exos was concluded to be the appropriate dosage of Exos for acute UC. This finding may provide a new dosage reference for the application of Exos in acute UC.

## 5. Conclusions

Our findings confirm that HFMSCs exert therapeutic effects against DSS-induced UC and pyroptosis by releasing Exos in a paracrine mechanism. The evidence obtained with HFMSCs, as novel advantageous MSCs, may provide new insights for research on MSCs in UC. Based on the bioinformatics results, pyroptosis is supposed to be a potential target of Exos for the treatment of UC.

## Data Availability

All the data obtained and analysed during this study are shown in this article.

## Abbreviations

UC: Ulcerative colitis
MSC: Mesenchymal stem cell
HFMSC: Hair follicle-derived mesenchymal stem cell
Exo: Exosome
NOD: Nucleotide oligomerization domain
DSS: Dextran sulfate sodium
NLRP3: NOD-like receptor pyrin domain-containing protein 3
ASC: Apoptosis-associated speck-like protein containing a caspase recruitment domain
5-ASA: 5-Aminosalicylic acid
GSDMD: Gasdermin D
FBS: Foetal bovine serum
IL-1*β*: Interleukin 1*β*
IL-18: Interleukin 18
CK15: Cytokeratin 15
DAI: Disease activity index
TEM: Transmission electron microscopy
HE: Haematoxylin and eosin
NTA: Nanoparticle tracking analysis
LPS: Lipopolysaccharide
ATP: Adenosine 5-triphosphate
SDS–PAGE|: Sodium dodecyl sulfate–polyacrylamide gel electrophoresis
CCK-8: Cell counting kit-8
EdU: 5-Ethynyl-2′-deoxyuridine
GEO: Gene Expression Omnibus
GO: Gene Ontology
KEGG: Kyoto Encyclopedia of Genes and Genomes
CC: Cellular component
MF: Molecular function
BP: Biological process
ANOVA: Analysis of variance
PCNA: Proliferating cell nuclear antigen
TRAIL: Tumour necrosis factor-related apoptosis-inducing ligand
IFN: Interferon
RIPK1: Receptor-interacting protein kinase 1
mTOR: Mechanistic target of rapamycin
TXNIP: Thioredoxin-interacting protein
DiR: 1,1′-Dioctadecyl-3,3,3′,3′-tetramethylindotricarbocyanine iodide.

## Supplementary Materials

Supplementary Materials Figure S1: HFMSCs downregulated the expression of pyroptosis-related proteins. Figure S2: the culture supernatant of HFMSC inhibited the levels of pyroptosis-related proteins in MODE-K cells induced by LPS+ATP. Figure S3: *in vitro* effect of HFMSC-released Exos on pyroptosis. Figure S4: HFMSC-derived Exos suppressed the expression of pyroptosis-related proteins on the colon. Table S1: primary antibodies used in the experiments. Table S2: DEmiRNAs in the GSE71241 dataset.
