# Supplementary material for: From Hair to Colon: Hair Follicle-Derived MSCs Alleviate Pyroptosis in DSS-Induced Ulcerative Colitis by Releasing Exosomes in a Paracrine Manner
Source: Oxid Med Cell Longev. 2022 Sep 16;2022:9097530. doi: 10.1155/2022/9097530 (PMC9507792; doi:10.1155/2022/9097530)
Supplement: Supplementary Materials — Figure S1: HFMSCs downregulated the expression of pyroptosis-related proteins. Figure S2: the culture supernatant of HFMSC inhibited the levels of pyroptosis-related proteins in MODE-K cells induced by LPS+ATP. Figure S3: in vitro effect of HFMSC-released Exos on pyroptosis. Figure S4: HFMSC-derived Exos suppressed the expression of pyroptosis-related proteins on the colon. Table S1: primary antibodies used in the experiments. Table S2: DEmiRNAs in the GSE71241 dataset. [file 9097530.f1.docx]

## Supplemental experimental procedures

**Extraction and characterization of Exos**

HFMSCs were plated in exosome-free culture medium with DMEM/F12 exosome-depleted serum for 24-48 h. Through differential centrifugation [1], Exos were isolated and purified from the supernatant of the HFMSC culture medium. The morphology and particle size of Exos were determined using transmission electron microscopy (TEM) and nanoparticle tracking analysis (NTA). Exo surface marker proteins, including the positive marker proteins CD9 (1:1000, Abcam) and TSG101 (1:1500, Abcam) and the negative marker protein calnexin (1:1500, Abcam), were detected by Western blotting.

## **References**

## [1] H. S. Han, H. Lee, D. You et al., "Human adipose stem cell-derived extracellular nanovesicles for treatment of chronic liver fibrosis," *Journal of controlled release : official journal of the Controlled Release Society*, vol. 320, pp. 328-336, 2020.

## Supplemental items

**Figure S1. HFMSCs downregulated the expression of pyroptosis-related proteins.**

Western blotting was carried out to examine the expression of NLRP3, GSDMD, cleaved-caspase-1 and IL-1β proteins. Semi-quantitative analysis of NLRP3, GSDMD, cleaved-caspase-1 and IL-1β proteins levels. All the data are presented as the means ± SDs. DSS+PBS *VS* DSS+HFMSC，* *P*<0.05. ** *P*<0.01.


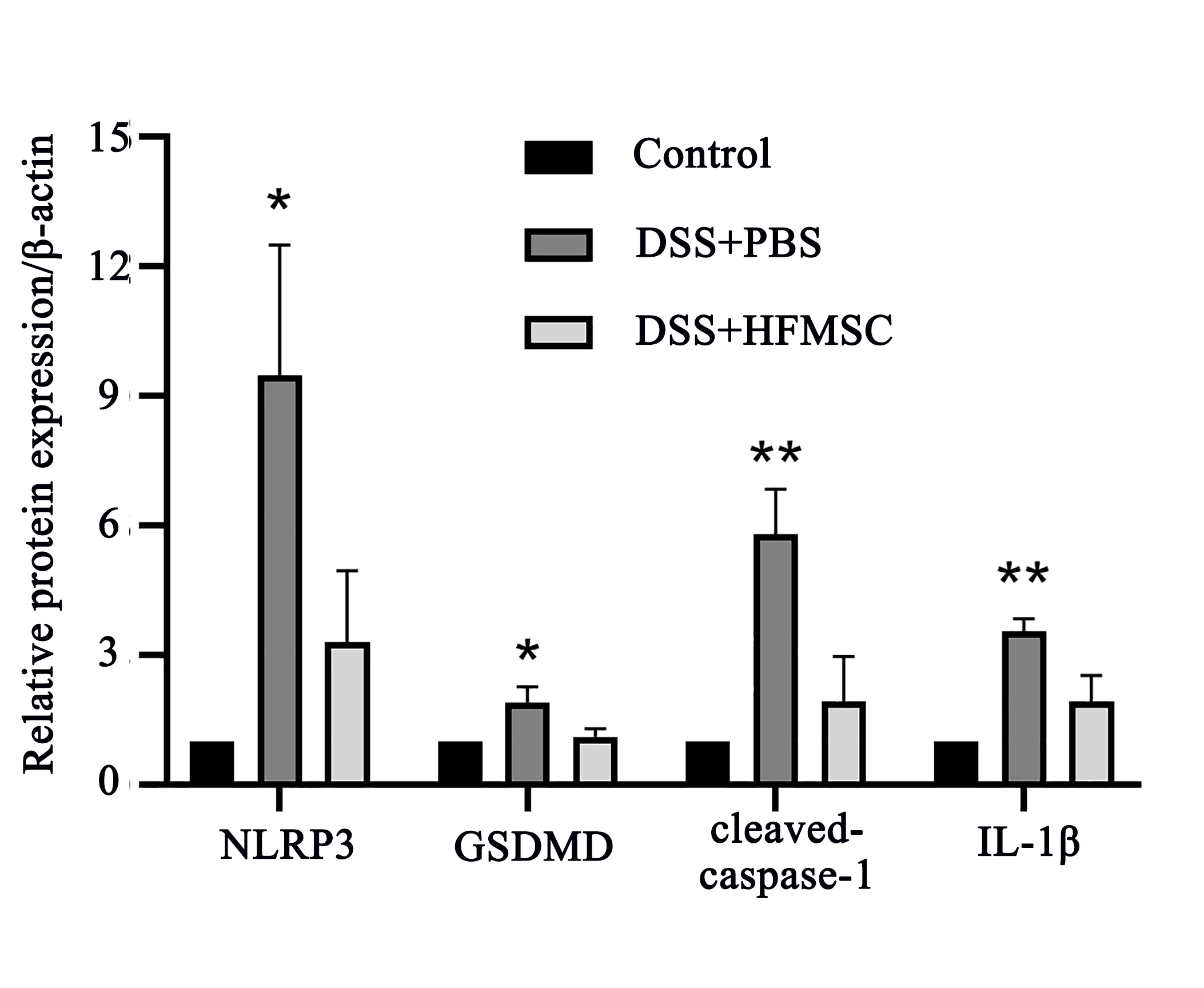


**Figure S2. The culture supernatant of HFMSC inhibited the levels of pyroptosis-related proteins in MODE-K cells induced by LPS+ATP.**

The expression of NLRP3, GSDMD, Cleaved caspase-1 and IL-1β proteins were detected by Western blotting. Statistical analysis of NLRP3, GSDMD, Cleaved caspase-1, IL-1β protein expression levels. All the data are presented as the means ± SDs. LPS+ATP+HFMSC / HFMSC+GW4869 *VS* LPS+ATP * *P*<0.05. # *P*<0.01. ** *P*<0.01.*** *P*<0.001.**** *P*<0.0001.

**
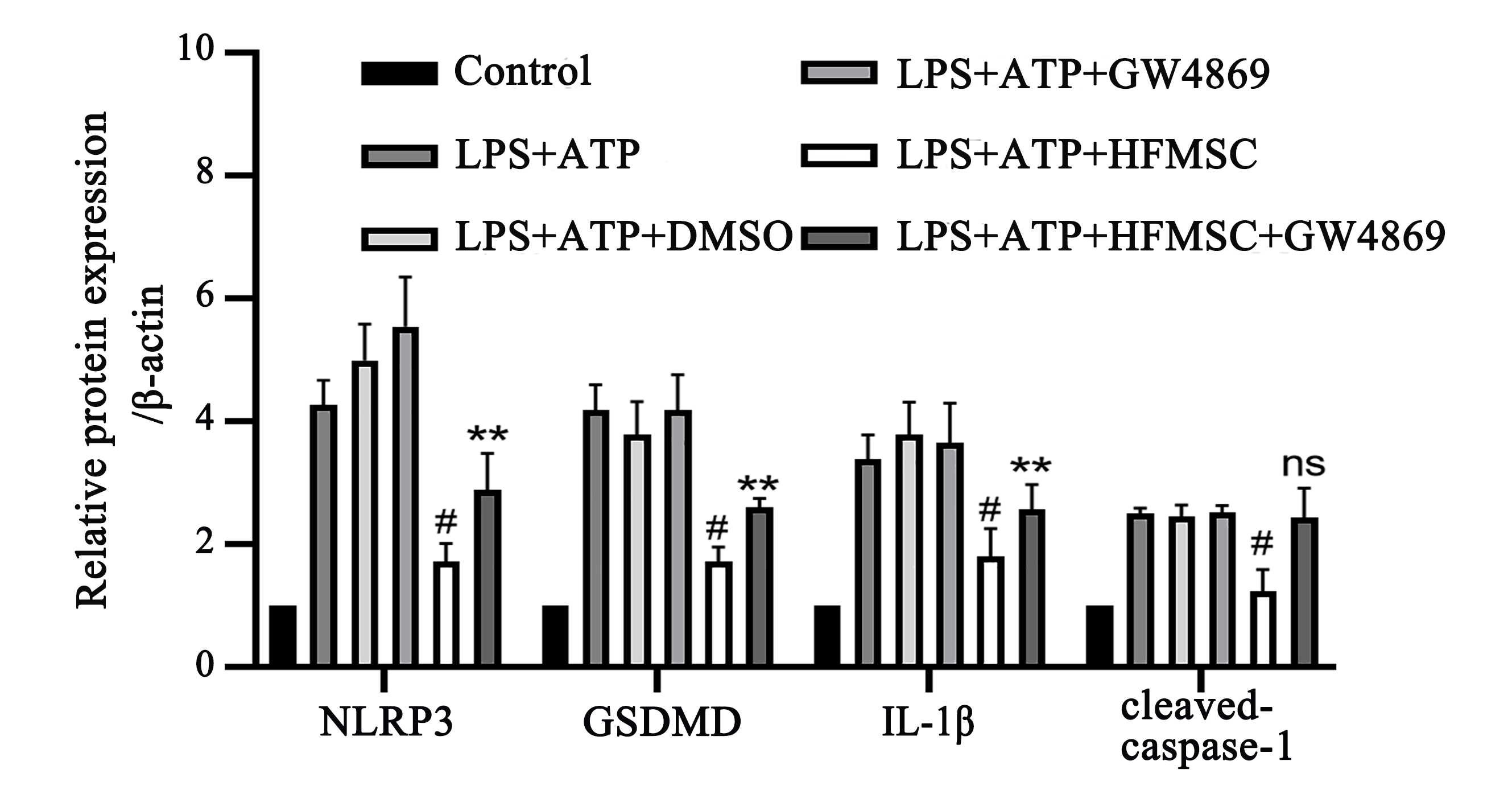
**

**Figure S3. *In vitro* effect of HFMSC-released Exos on pyroptosis**

Exos were extracted from the supernatant of HFMSCs in exosome-free culture medium by differential centrifugation. [A-B] The appearance and particle size of Exos were analysed by TEM and NTA. [C] Western blotting detect the expression of the positive surface markers CD9 and TSG101 and the negative surface marker calnexin on Exos. After the incubation of PKH67-dyed Exos with MODE-K cells for 24 h, [D] the uptake of Exos by MODE-K cells was detected through immunofluorescence. Scale bar, 100 µm. Subsequently, 100 μg/ml Exos were incubated with MODE-K cells stimulated with LPS and ATP. [E-G] EdU and CCK-8 were determined to assess the cell viability in different groups. Scale bar, 200 µm. In addition, the impact of Exos on pyroptosis was verified by [H-L] Western blotting and [M-N] ELISA. All the data are shown as the means ± SDs. * *P*<0.05. ** *P*<0.01. *** *P*<0.001.


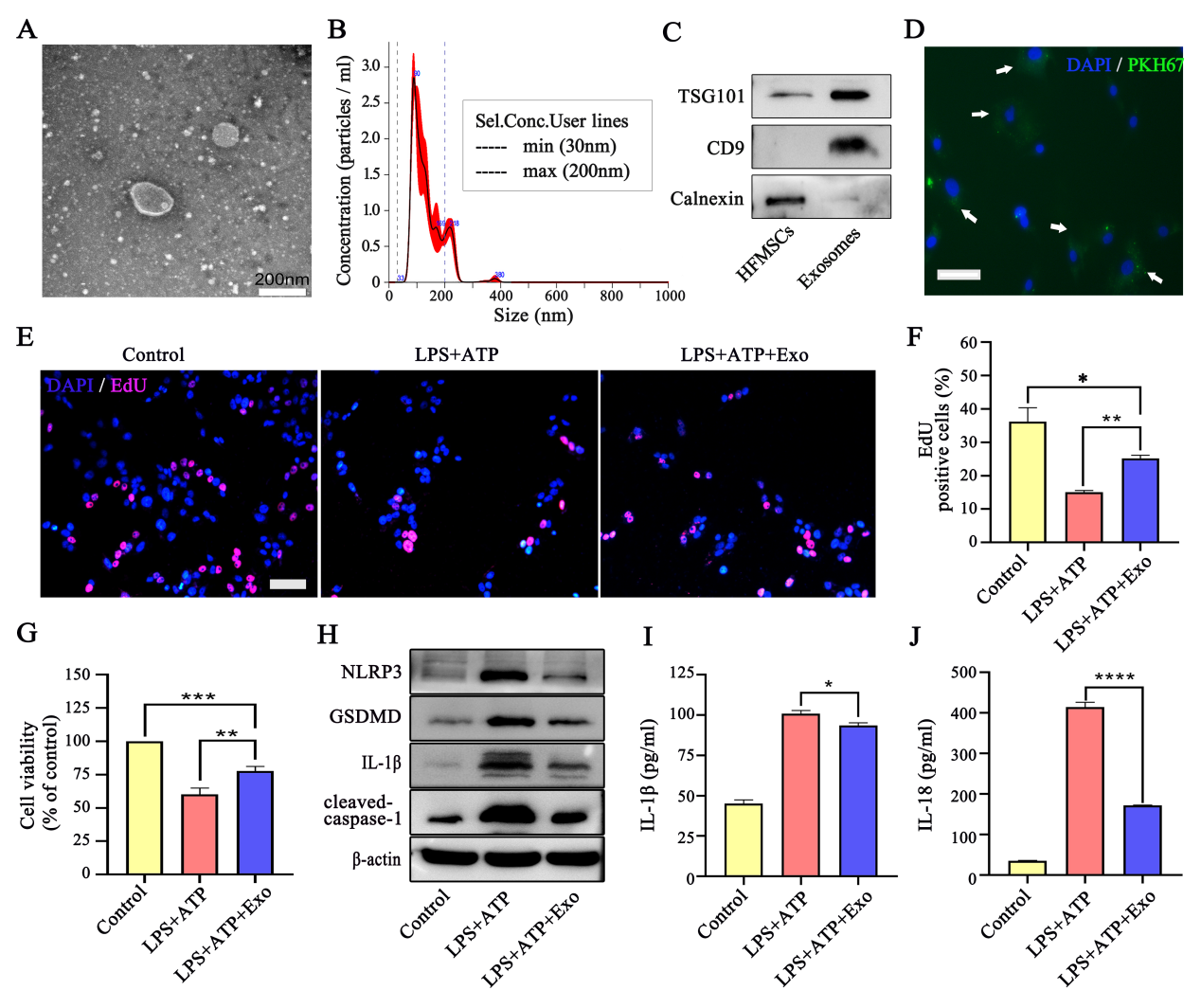


**Figure S4. HFMSC-derived Exos suppressed the expression of pyroptosis-related proteins on the colon.**

Western blotting was carried out to examine the expression of NLRP3, GSDMD, cleaved-caspase-1 and IL-1β proteins. Semi-quantitative analysis of NLRP3, GSDMD, cleaved-caspase-1 and IL-1β proteins levels. All the data are presented as the means ± SDs. DSS+Exo *VS* DSS+PBS ** *P*<0.01. ## *P*<0.0001.


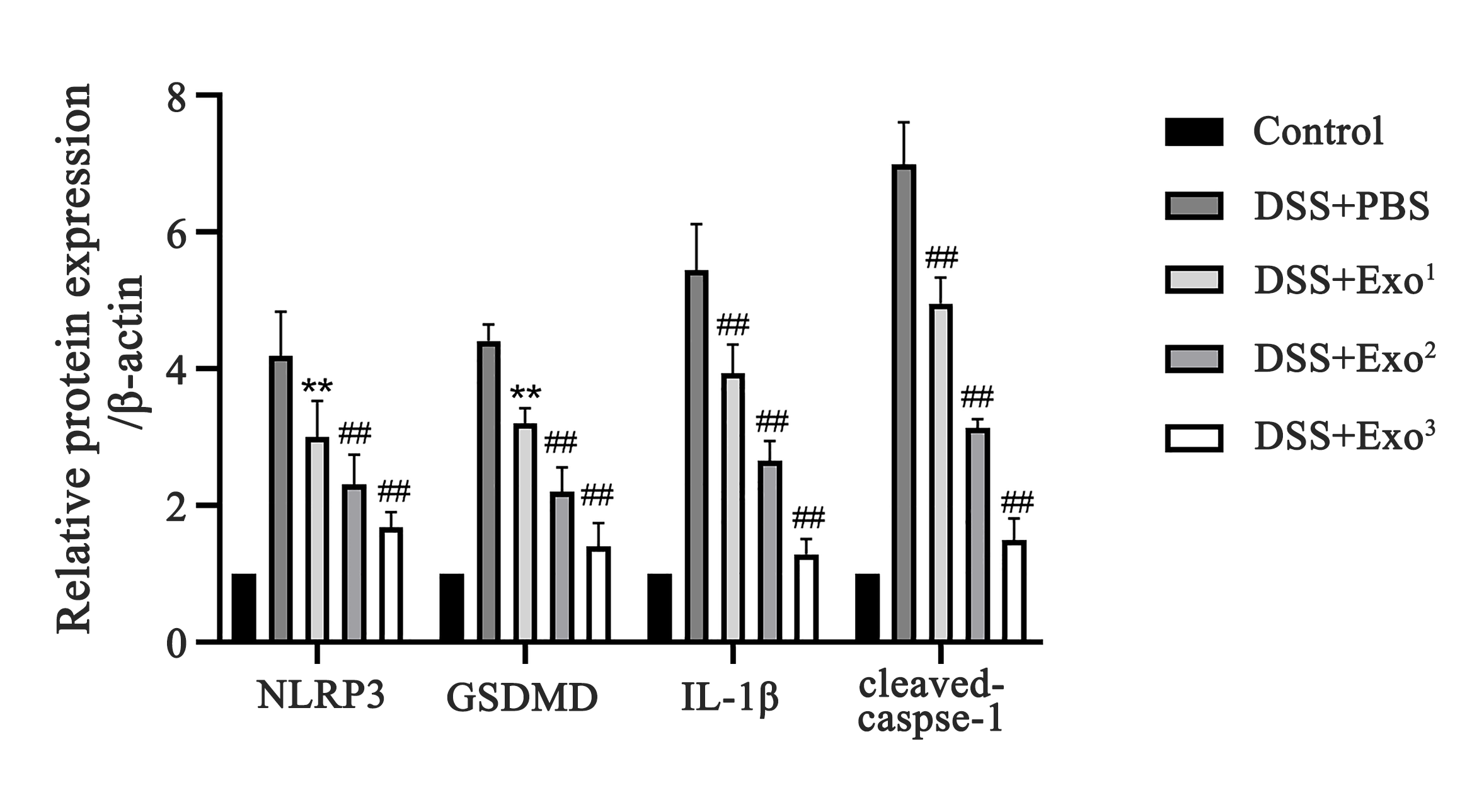


**Table S1. Primary-antibodies used in the experiments**

| **Antibody** | **Experimental application and dilution** | **Company** |
| --- | --- | --- |
| **Anti-mouse/rat CD29** | Flow cytometry: 1 µg/test | Thermo Fisher |
| **Anti-mouse/rat CD90** | Flow cytometry: 1 µg/test | Thermo Fisher |
| **Anti-mouse/rat CD31** | Flow cytometry: 5 µg/test | Thermo Fisher |
| **Anti-mouse/rat CD43** | Flow cytometry: 0.5 µg/test | Thermo Fisher |
| **Rabbit anti-mouse CK15** | Immunofluorescence (1:500) | Santa Cruz |
| **Rabbit anti-mouse PCNA** | Immunofluorescence (1:200) | Affinity |
| **Rabbit anti-mouse NLRP3** | Western blotting (1:5000) Immunofluorescence (1:50) | NOVUS |
| **Rabbit anti-mouse GSDMD** | Western blotting (1:1000) Immunofluorescence (1:1000) | Abcam |
| **Rabbit anti-mouse cleaved- caspase-1** | Western blotting (1:1000) | Cell Signalling Technology |
| **Rabbit anti-mouse IL-1β** | Western blotting (1:1000) | ABclonal |
| **Rabbit anti-mouse β-actin** | Western blotting (1:1000) | Abcam |
| **Rabbit anti-mouse CD9** | Western blotting (1:1000) | Abcam |
| **Rabbit anti-mouse TSG101** | Western blotting (1:1500) | Abcam |
| **Rabbit anti-mouse calnexin** | Western blotting (1:1500) | Abcam |

**Table S2. DE-miRNAs in GSE71241 dataset**

| **Symbol** | **logFC** | **P.Value** | **adj.P.Val** | **change** |
| --- | --- | --- | --- | --- |
| hsa-miR-451 | 9.001130878 | 9.61E-06 | 8.49E-05 | UP |
| bkv-miR-B1-5p | 1.008929223 | 0.002576875 | 0.00332568 | UP |
| hsa-miR-142-3p | 3.738265956 | 0.004148016 | 0.005317105 | UP |
| hsa-miR-513c | 1.0028977 | 0.004294131 | 0.005489524 | UP |
| hsa-miR-142-5p | 1.046030538 | 0.0044669 | 0.005679687 | UP |
| hsa-miR-122 | 1.048063191 | 0.004589016 | 0.005827126 | UP |
| hsa-miR-30c-2* | 1.051785862 | 0.004817684 | 0.006109288 | UP |
| hsa-miR-422a | 1.053395963 | 0.004918595 | 0.006228903 | UP |
| hsa-miR-520e | 1.477953878 | 0.005047975 | 0.006384204 | UP |
| hsa-miR-520b | 1.056252238 | 0.005100589 | 0.006442132 | UP |
| hsa-miR-634 | 3.195467956 | 0.00513997 | 0.006483215 | UP |
| hsa-miR-144 | 4.319853856 | 0.005164032 | 0.006504893 | UP |
| hsa-miR-877* | 1.220166756 | 0.005701967 | 0.007172953 | UP |
| hsa-miR-1249 | 1.286799811 | 0.006766326 | 0.008478073 | UP |
| hsa-miR-1308 | 3.587011002 | 0.00911887 | 0.011320802 | UP |
| hsa-miR-1268 | 3.512056168 | 0.010281853 | 0.012747881 | UP |
| hsa-miR-223 | 2.651883122 | 0.010346191 | 0.01281086 | UP |
| hsa-miR-345 | 1.733592333 | 0.010376672 | 0.012831806 | UP |
| hsa-miR-671-5p | 4.320562511 | 0.010733275 | 0.013255455 | UP |
| hsa-miR-601 | 1.779447078 | 0.011868343 | 0.014562195 | UP |
| hsa-miR-877 | 1.249292644 | 0.012282902 | 0.015031857 | UP |
| hsa-miR-1471 | 1.832861622 | 0.013252007 | 0.016175998 | UP |
| hsa-miR-1226* | 1.467791595 | 0.013659474 | 0.016651885 | UP |
| hsa-miR-583 | 1.156866878 | 0.013781319 | 0.0167788 | UP |
| hsa-miR-622 | 1.333925356 | 0.014051692 | 0.017064057 | UP |
| hsa-miR-1246 | 3.281507313 | 0.014806065 | 0.017934107 | UP |
| ebv-miR-BHRF1-1 | 3.05226534 | 0.014950748 | 0.018063101 | UP |
| hsa-miR-1183 | 1.168317911 | 0.015015843 | 0.018118606 | UP |
| hsv1-miR-H1 | 1.871098167 | 0.015127469 | 0.018230045 | UP |
| hsa-miR-623 | 1.880766402 | 0.01780957 | 0.021380524 | UP |
| hsa-miR-885-3p | 1.0885925 | 0.018273996 | 0.021910266 | UP |
| hsa-miR-498 | 1.608480844 | 0.019063064 | 0.022798557 | UP |
| hsa-miR-150 | 1.207753867 | 0.019567603 | 0.023372414 | UP |
| hcmv-miR-US4 | 1.440542611 | 0.019645243 | 0.023435561 | UP |
| hsa-miR-1539 | 1.106466689 | 0.020540948 | 0.02447322 | UP |
| hsa-miR-129-3p | 1.7331767 | 0.021517104 | 0.025604001 | UP |
| hsa-miR-1290 | 3.962621652 | 0.02274555 | 0.026997855 | UP |
| hsa-miR-1914* | 2.894352933 | 0.023902357 | 0.028299913 | UP |
| hsa-miR-129* | 1.1375581 | 0.024685154 | 0.029190195 | UP |
| hsa-miR-513b | 1.028505622 | 0.025583778 | 0.030215048 | UP |
| hsa-miR-33b* | 3.271866678 | 0.027049202 | 0.031905917 | UP |
| kshv-miR-K12-3 | 3.019978947 | 0.027236038 | 0.032086291 | UP |
| hsa-miR-1225-3p | 2.845956172 | 0.028566114 | 0.03356962 | UP |
| hsa-miR-765 | 1.735747544 | 0.028693253 | 0.033677192 | UP |
| hsa-miR-766 | 2.607242536 | 0.028956283 | 0.033943797 | UP |
| hsa-miR-572 | 2.836451467 | 0.0315858 | 0.036934694 | UP |
| hsa-miR-638 | 3.153951244 | 0.034949521 | 0.040740454 | UP |
| hsa-let-7b* | 1.934770889 | 0.034969608 | 0.040740454 | UP |
| hsa-miR-1305 | 1.405680011 | 0.035746288 | 0.041594081 | UP |
| hsa-miR-1202 | 3.344882562 | 0.036389961 | 0.042291036 | UP |
| hsa-miR-1234 | 2.935685712 | 0.038075789 | 0.044141785 | UP |
| hsa-miR-630 | 3.299839944 | 0.038464774 | 0.044538159 | UP |
| hsa-miR-191* | 2.859840717 | 0.040700959 | 0.047069813 | UP |
| hsa-miR-1228 | 2.925224024 | 0.041335109 | 0.047744827 | UP |
| hsa-miR-486-5p | 1.377652844 | 0.042345701 | 0.048733617 | UP |
| hsa-miR-210 | -4.799045911 | 1.76E-19 | 1.66E-16 | DOWN |
| hsa-miR-377 | -4.364475522 | 5.40E-18 | 2.55E-15 | DOWN |
| hsa-miR-10a | -3.977246011 | 2.48E-17 | 7.81E-15 | DOWN |
| hsa-miR-31* | -4.012900133 | 5.33E-17 | 1.26E-14 | DOWN |
| hsa-miR-193a-5p | -3.767554507 | 1.07E-15 | 2.03E-13 | DOWN |
| hsa-miR-143 | -4.569077456 | 8.97E-15 | 1.41E-12 | DOWN |
| hsa-miR-574-3p | -3.864613737 | 1.29E-14 | 1.75E-12 | DOWN |
| hsa-miR-654-3p | -3.95986306 | 1.84E-14 | 2.17E-12 | DOWN |
| hsa-miR-196a | -3.79909498 | 2.18E-14 | 2.29E-12 | DOWN |
| hsa-miR-125a-5p | -4.489276533 | 3.07E-14 | 2.90E-12 | DOWN |
| hsa-miR-98 | -3.407718852 | 1.15E-13 | 9.93E-12 | DOWN |
| hsa-miR-140-5p | -6.946465089 | 1.41E-13 | 1.11E-11 | DOWN |
| hsa-miR-34a | -6.39893779 | 1.65E-13 | 1.20E-11 | DOWN |
| hsa-miR-31 | -4.272683578 | 2.49E-13 | 1.68E-11 | DOWN |
| ebv-miR-BART12 | -3.222124861 | 3.51E-13 | 2.22E-11 | DOWN |
| hsa-miR-493* | -3.674383391 | 1.62E-12 | 9.57E-11 | DOWN |
| hsa-miR-27b | -7.379360689 | 2.18E-12 | 1.21E-10 | DOWN |
| hsa-miR-196b | -3.213015687 | 2.82E-12 | 1.48E-10 | DOWN |
| hsa-let-7e | -6.933100233 | 4.27E-12 | 2.13E-10 | DOWN |
| hsa-miR-299-5p | -3.42506001 | 8.19E-12 | 3.88E-10 | DOWN |
| hsa-miR-199a-5p | -4.779504651 | 9.62E-12 | 4.33E-10 | DOWN |
| hsa-miR-29a | -7.700001778 | 1.16E-11 | 4.98E-10 | DOWN |
| hsa-miR-222 | -3.745457811 | 1.39E-11 | 5.71E-10 | DOWN |
| hsa-miR-365 | -6.663386356 | 2.25E-11 | 8.39E-10 | DOWN |
| hsa-miR-100 | -7.898652256 | 2.26E-11 | 8.39E-10 | DOWN |
| hsa-miR-29c | -4.610822093 | 2.31E-11 | 8.39E-10 | DOWN |
| hsa-miR-424 | -5.007082653 | 3.21E-11 | 1.12E-09 | DOWN |
| hsa-miR-99a | -4.113180378 | 3.68E-11 | 1.24E-09 | DOWN |
| hsa-miR-30a | -4.047907802 | 4.33E-11 | 1.35E-09 | DOWN |
| hsa-miR-214 | -4.400320122 | 4.36E-11 | 1.35E-09 | DOWN |
| hsa-let-7c | -7.196404511 | 4.43E-11 | 1.35E-09 | DOWN |
| hsa-miR-376a | -4.214915313 | 1.78E-10 | 5.25E-09 | DOWN |
| hsa-miR-10b | -4.0385853 | 1.94E-10 | 5.57E-09 | DOWN |
| hsa-miR-193b | -3.891220304 | 2.92E-10 | 8.13E-09 | DOWN |
| hsa-miR-376c | -4.227196458 | 3.31E-10 | 8.76E-09 | DOWN |
| hsa-miR-23b | -7.600989078 | 3.33E-10 | 8.76E-09 | DOWN |
| hsa-miR-136 | -5.87635402 | 3.60E-10 | 9.04E-09 | DOWN |
| hsa-miR-130a | -4.672646722 | 3.63E-10 | 9.04E-09 | DOWN |
| hsa-miR-140-3p | -4.080097959 | 4.10E-10 | 9.96E-09 | DOWN |
| hsa-miR-181a | -4.377701173 | 6.34E-10 | 1.50E-08 | DOWN |
| hsa-miR-103 | -4.584402538 | 8.97E-10 | 2.07E-08 | DOWN |
| hsa-miR-24 | -7.858717822 | 1.19E-09 | 2.68E-08 | DOWN |
| hsa-miR-193a-3p | -5.979269444 | 1.29E-09 | 2.85E-08 | DOWN |
| hsa-let-7b | -7.842088467 | 2.24E-09 | 4.82E-08 | DOWN |
| hsa-miR-29b | -6.457469522 | 2.60E-09 | 5.48E-08 | DOWN |
| hsa-let-7i | -7.003978889 | 3.32E-09 | 6.83E-08 | DOWN |
| hsa-miR-1274a | -4.05344087 | 3.69E-09 | 7.42E-08 | DOWN |
| hsa-miR-199a-3p | -6.850038433 | 3.82E-09 | 7.52E-08 | DOWN |
| hsa-miR-127-3p | -3.616278827 | 3.90E-09 | 7.52E-08 | DOWN |
| hsa-miR-432 | -2.613880171 | 4.91E-09 | 9.16E-08 | DOWN |
| hsa-miR-145 | -5.919760089 | 4.94E-09 | 9.16E-08 | DOWN |
| hsa-miR-221 | -6.796932533 | 7.08E-09 | 1.29E-07 | DOWN |
| hsa-let-7d | -4.6947439 | 7.66E-09 | 1.37E-07 | DOWN |
| hsa-miR-107 | -4.764256522 | 1.01E-08 | 1.77E-07 | DOWN |
| hsa-miR-26a | -4.683187944 | 1.13E-08 | 1.94E-07 | DOWN |
| hsa-miR-125b | -8.248225044 | 1.50E-08 | 2.53E-07 | DOWN |
| hsa-let-7g | -5.029701278 | 1.61E-08 | 2.68E-07 | DOWN |
| hsa-miR-320b | -3.9861337 | 1.97E-08 | 3.21E-07 | DOWN |
| hsa-miR-151-5p | -3.998876378 | 2.31E-08 | 3.70E-07 | DOWN |
| hsa-miR-320d | -4.3598356 | 2.99E-08 | 4.72E-07 | DOWN |
| hsa-miR-15a | -4.485728122 | 7.97E-08 | 1.24E-06 | DOWN |
| hsa-miR-23a | -8.1581791 | 8.96E-08 | 1.37E-06 | DOWN |
| hsa-miR-27a | -7.120927 | 1.28E-07 | 1.92E-06 | DOWN |
| hsa-miR-1274b | -6.240962067 | 2.15E-07 | 3.18E-06 | DOWN |
| hsa-miR-16 | -4.888292933 | 2.25E-07 | 3.28E-06 | DOWN |
| hsa-miR-26b | -4.291509889 | 2.56E-07 | 3.66E-06 | DOWN |
| hsa-let-7f | -7.370813722 | 6.61E-07 | 8.22E-06 | DOWN |
| hsa-miR-19b | -3.830717533 | 7.51E-07 | 9.11E-06 | DOWN |
| hsa-miR-137 | -3.6119136 | 1.26E-06 | 1.41E-05 | DOWN |
| hsa-miR-379 | -3.284831291 | 1.89E-06 | 1.98E-05 | DOWN |
| hsa-miR-455-3p | -3.103644209 | 2.07E-06 | 2.13E-05 | DOWN |
| hsa-miR-337-5p | -2.747760202 | 2.31E-06 | 2.30E-05 | DOWN |
| hsa-miR-152 | -2.712846772 | 2.85E-06 | 2.75E-05 | DOWN |
| hsa-miR-28-5p | -2.858492523 | 3.16E-06 | 2.96E-05 | DOWN |
| hsa-miR-221* | -2.87100818 | 4.49E-06 | 4.16E-05 | DOWN |
| hsa-miR-574-5p | -2.936531602 | 5.18E-06 | 4.75E-05 | DOWN |
| hsa-miR-15b | -3.746419356 | 5.73E-06 | 5.21E-05 | DOWN |
| hsa-miR-99b | -2.514542293 | 6.68E-06 | 6.02E-05 | DOWN |
| hsa-miR-382 | -1.935729135 | 1.18E-05 | 0.000103174 | DOWN |
| hsa-miR-335 | -2.375385933 | 1.36E-05 | 0.000117623 | DOWN |
| hsa-miR-30a* | -2.186792237 | 1.84E-05 | 0.000158439 | DOWN |
| hsa-let-7a | -6.6178502 | 2.02E-05 | 0.00017192 | DOWN |
| hsa-miR-185 | -2.13047318 | 3.41E-05 | 0.000214637 | DOWN |
| hsa-miR-409-3p | -2.239086606 | 4.80E-05 | 0.000214637 | DOWN |
| hsa-miR-361-5p | -2.265589485 | 5.66E-05 | 0.000214637 | DOWN |
| hsa-miR-30d | -2.641449014 | 8.65E-05 | 0.000214637 | DOWN |
| hsa-miR-30e | -2.13089896 | 0.000101399 | 0.000214637 | DOWN |
| hsa-miR-1185 | -2.12788182 | 0.000124628 | 0.000214637 | DOWN |
| hsa-miR-21 | -7.423571156 | 0.000170822 | 0.000233522 | DOWN |
| hsa-miR-30c | -2.045798368 | 0.000172214 | 0.000235086 | DOWN |
| hsa-miR-34b* | -2.4774661 | 0.000273357 | 0.000366802 | DOWN |
| miRNABrightCorner30 | -5.272518 | 0.00028195 | 0.000377797 | DOWN |
| hsa-miR-337-3p | -2.102050939 | 0.000404337 | 0.000541023 | DOWN |
| hsa-miR-376a* | -1.945794892 | 0.000513799 | 0.000686517 | DOWN |
| hsa-miR-24-1* | -1.88750508 | 0.000601853 | 0.000800778 | DOWN |
| hsa-miR-22 | -5.421300433 | 0.000627111 | 0.000830878 | DOWN |
| hsa-miR-485-3p | -1.774751777 | 0.000758813 | 0.001001168 | DOWN |
| hsa-miR-224 | -1.996454656 | 0.000908382 | 0.001193512 | DOWN |
| hsa-miR-495 | -2.126057912 | 0.001112105 | 0.001457135 | DOWN |
| hsa-miR-410 | -1.716589785 | 0.001187402 | 0.001551495 | DOWN |
| hsa-miR-331-3p | -3.211849493 | 0.001311051 | 0.001705989 | DOWN |
| hsa-miR-758 | -1.555501452 | 0.001527423 | 0.001984811 | DOWN |
| hsa-miR-148a | -2.315686401 | 0.001788325 | 0.002320652 | DOWN |
| hsa-miR-30b | -1.60542354 | 0.001837713 | 0.002381475 | DOWN |
| hsa-miR-30e* | -1.526293432 | 0.002003249 | 0.002592439 | DOWN |
| hsa-miR-720 | -4.898405644 | 0.002649131 | 0.003414275 | DOWN |
| hsa-miR-301a | -1.678234347 | 0.003670763 | 0.004711725 | DOWN |
| hsa-miR-886-3p | -2.352011106 | 0.004426342 | 0.005635693 | DOWN |
| hsa-miR-195 | -1.694076856 | 0.007244055 | 0.009064651 | DOWN |
| hsa-miR-20a | -1.892377627 | 0.00843121 | 0.010522328 | DOWN |
| hsa-miR-324-5p | -1.471079344 | 0.008675749 | 0.010813252 | DOWN |
| hsa-miR-106b | -1.769243471 | 0.010847538 | 0.013379102 | DOWN |
| hsa-miR-487b | -1.36890617 | 0.011148881 | 0.013715009 | DOWN |
| hsa-miR-660 | -1.19068395 | 0.011916478 | 0.014602317 | DOWN |
| hsa-miR-186 | -1.16758002 | 0.013970259 | 0.016986973 | DOWN |
| hsa-miR-22* | -1.85265021 | 0.01409265 | 0.017091855 | DOWN |
| hsa-miR-342-3p | -1.07605496 | 0.014914262 | 0.018042061 | DOWN |
| hsa-miR-151-3p | -1.223838171 | 0.015382702 | 0.018514041 | DOWN |
| hsa-miR-19a | -1.50147998 | 0.029339853 | 0.034350867 | DOWN |
| hsa-miR-320a | -1.536821492 | 0.033454959 | 0.039072088 | DOWN |
| hsa-miR-1260 | -2.404658111 | 0.036513407 | 0.042382433 | DOWN |
| hsa-miR-92a | -1.583962651 | 0.041386007 | 0.047745321 | DOWN |
